# Memory of chirality in a room temperature flow electrochemical reactor

**DOI:** 10.1038/s41598-020-73957-6

**Published:** 2020-10-06

**Authors:** Tomas Hardwick, Rossana Cicala, Thomas Wirth, Nisar Ahmed

**Affiliations:** 1 School of Chemistry, Cardiff University, Main Building, Park Place, Cardiff, CF10 3AT UK; 2 National Graphene Institute, University of Manchester, Oxford Road, Manchester, M13 9PL UK; 3 International Centre for Chemical and Biological Sciences, HEJ Research Institute of Chemistry, University of Karachi, Karachi, 75270 Pakistan

**Keywords:** Chemistry, Catalysis, Chemical engineering, Electrochemistry, Green chemistry, Organic chemistry

## Abstract

Chiral compounds have become of great interest to the pharmaceutical industry as they possess various biological activities. Concurrently, the concept of “memory of chirality” has been proven as a powerful tool in asymmetric synthesis, while flow chemistry has begun its rise as a new enabling technology to add to the ever increasing arsenal of techniques available to the modern day chemist. Here, we have employed a new simple electrochemical microreactor design to oxidise an l-proline derivative at room temperature in continuous flow. Compared to batch, organic electrosynthesis via microflow reactors are advantageous because they allow shorter reaction times, optimization and scale up, safer working environments, and high selectivities (e.g. reduce overoxidation). Flow electrochemical reactors also provide high surface-to-volume ratios and impart the possibility of excluding the supporting electrolyte due to a very short interelectrode distance. By the comparison of Hofer Moest type electrochemical oxidations at room temperature in batch and flow, we conclude that continuous flow electrolysis is superior to batch, producing a good yield (71%) and a higher enantiomeric excess (64%). These results show that continuous flow has the potential to act as a new enabling technology for asymmetric synthesis to replace some aspects of conventional batch electrochemical processes.

## Introduction

Processes in batch are dependent on the concentration of reagents, their volume within the vessel and the time at which the reaction is performed at a given temperature. Processes in flow are dependent on the concentration of reagents, their flow rates, the reactor volume and the bulk flow rate^1,2^. The optimal flow condition is that which allows full conversion to be realised with the highest possible flow rate. Flow allows the reaction mixture to flow through channels where they can be intercepted at junctions, heated, cooled or irradiated before leaving the reactor^3^. Reactions will continue until reagents are no longer introduced, thus allowing scale to become dependent on time rather than vessel volume, and allows easy access to high pressure reactions (by addition of a back pressure regulator). Because of the ease of control of flow parameters it is not surprising that significant advancements in reactor technology are on the rise^4–11^.

By gaining a greater understanding of the control parameters, heat transfer, mass transport and efficient mixing, recent chemical engineering progress in flow has introduced the concept of device miniaturisation in an attempt to manipulate chemical systems with difficult-to-handle reagents in an improved manner, in comparison to the conventional batch methodology^2,12^. Such improvements can be relayed in terms of a greater amount of a desired product per unit time^12^, swift reactions that may or may not involve multiple phases of matter to be run uninterrupted, a reduced exposure of harmful chemicals to the human operators. Moreover, fewer quantities of materials are required for screening, and the user is ultimately gifted with an enhanced control over selectivity, efficiency and thus, synthesises can be performed with a more green attribute^12^. Because the distance between the electrodes is so small (μm range), electrogenerated ions from the solvent are formed and can act as the systems electrolyte. This is due to the diffusion layers of the two closely positioned electrodes being able to couple^12–14^. In addition, the small diameters of the channels enable a high heat transfer capacity which is more desirable for micro, rather than mini, flow reactors^15^. With that being said, there are some difficulties that arise with a flow setup which include limited flow rates, dismantling and cleaning, problems dealing with solids within the tubes which can lead to blockages, high drops in pressure, and reaction screening due to the complexity of the setup^15,16^. It is, therefore, a necessity for microreactor technology to have reaction conditions optimised^16–27^.

**Figure 1:**
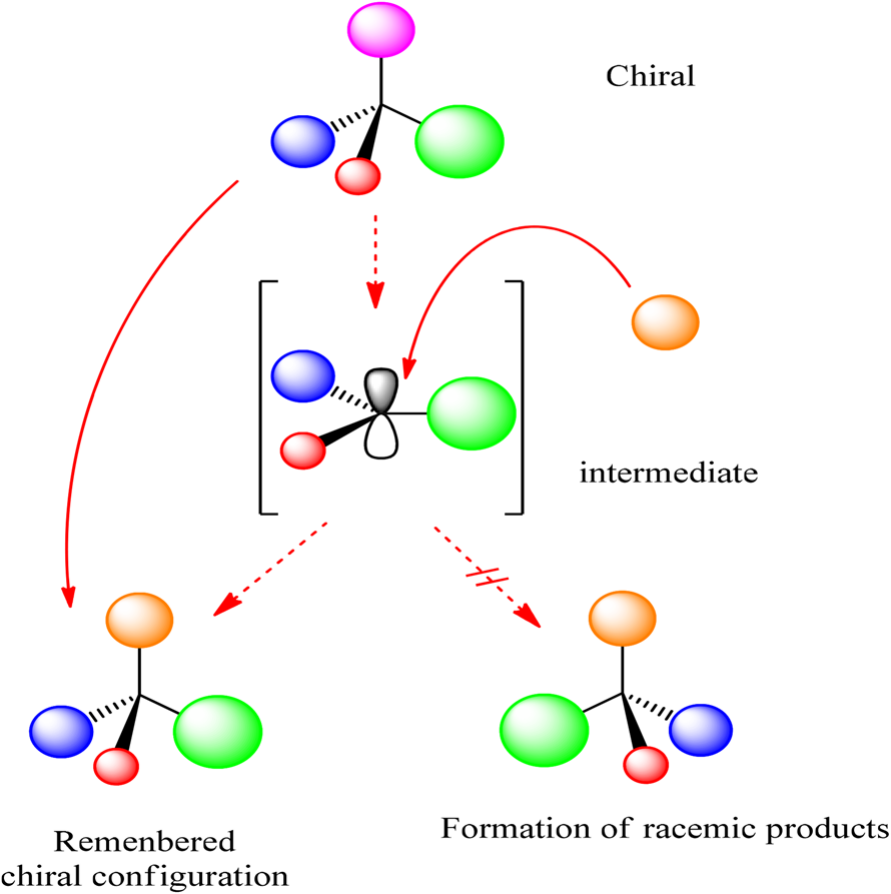
Racemic product formation from an enantiopure starting material.

It is known that trigonalising an enantiopure *sp*^3^ hybridised centre to *sp*^2^ will be yield products in an equal/racemic mixture, in the absence of any external factors (i.e. chiral auxiliaries). Nevertheless, there exists a phenomena known as “memory of chirality” (MOC) where, under three specific conditions allows, to a certain extent, the original stereochemistry to be retained in the product species, as illustrated in Fig. 1^28^. The conditions that must be met are: (i) an intermediate chiral species must be formed at the stereogenic centre of the initial material in an enantioselective manner^29^, (ii) this chiral intermediate must not readily racemise, and (iii) the chiral intermediate must react with high stereospecificity—show enhanced reactivity towards product formation compared to racemisation.

Memory of chirality (MOC) is quite a large topic which occurs through an enolate, anion, radical or cation intermediate. Here, we are only focused on the cationic intermediate case. The compound synthesised in this work is based on a derivative of l-proline with an amide functionality; such compounds have gained interest due to their involvement in the structures of complex artificial and natural products, such as proteins, pharmacological compounds and in biological processes such as enzyme catalysis, hemoglobin and antibodies. Peptides, a very common form of proline derivative, have a variety of uses. For example, Gly-Pro-Glu has neurological protection activity and may have involvement in the central nervous system (CNS)^29^ and Morphiceptin has found usage as a selective μ-opioid receptor agonist^30^. Such compounds can also be found in drugs such as Captopril which can be applied as an angiotensin-converting-enzyme (ACE) inhibitor, reliever of high blood pressure, congestive heart failure, kidney problems caused by diabetes and to improve survival after a heart attack^30^.

Recently, the use of compounds containing proline derivatives has been extended to the synthesis of natural products whilst combining with the concept of MOC^28^. In 2015, proline was used as the only chiral source in the synthesis of the natural marine alkaloid penibruguieramine A (PA) by Kim et al.^31^. In 2018 Hu and co-workers then extended this approach in order to synthesise the fluorine analogues of PA, 6 (*R*)- and 6 (*S*)-fluoropenibruguieramine^32^. Lately, Mambrini et al. have combined the concepts of MOC, microreactor flow chemistry, as well as flash chemistry to afford the large scale synthesis of l-alanine via enolate alkylation using 2.4 equiv. LDA and 5 equiv. Allyl iodide in THF with residence times of less than three minutes being reported^33^. High conversions and enantiomeric excess values were achieved in their flow system and were comparable to their batch counterparts, however, reaction temperatures used in flow had to be similar to that of batch, ranging from − 50 to − 78 °C. In 2019, MOC was extended to the synthesis of (R)-Boc-2-methylproline, a precursor to the Poly(ADP-ribose) Polymerase Inhibitor, veliparib^34^. The authors were able to produce several kilograms of the precursor in 99.6% ee (64% yield) in four steps without the use of chromatography, which was then further treated in three more steps to afford veliparib in 66% yield. It is clear that MOC is an interesting and powerful tool in asymmetric synthesis and we have henceforth taken inspiration from this phenomena, along with the requirement for the production of proline derivatives, and have expanded this motivation to the use of flow electrochemistry at room temperature.

**Figure 2:**
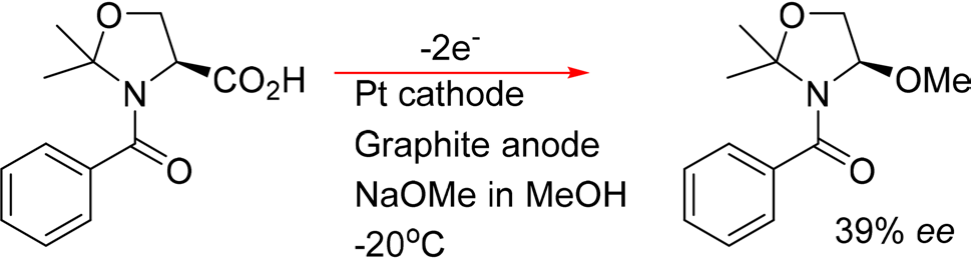
Electrochemical oxidation of an N-benzoylated serine derivative.

**Figure 3:**
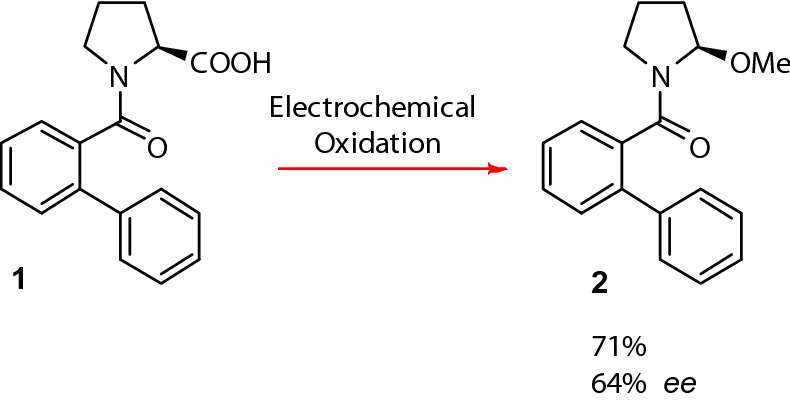
Electrochemical oxidation of ([1, 1′-biphenyl]-2-carbonyl)-l-proline (**1**) to proline derivative (R)-[1, 1′-biphenyl]-2-yl (2-methoxypyrrolidin-1-yl) methanone (**2**).

The first example of cationic memory of chirality was reported in 2000 by Matsumura et al., who used the batch electrochemical oxidation of an *N*-benzoylated serine derivative in methanol to give an *N*, *O*-acetal (Fig. 2) at − 20 °C^34^. They noted, however, that the enantiomeric excess of their reactions were generally quite low. Similarly, the same group replaced the l-serine for an l-proline component (**1**), which, under optimised conditions, afforded an enantiomeric excess of 46%^35^. With the aforementioned points highlighting the appeal of proline derivatives and MOC we have taken this reaction as an example, corresponding to the transformation of **1** into **2** (illustrated in Fig. 3), which did not perform satisfactory in batch, and have subjected it to continuous flow where the electrochemistry has be carried out at room temperature in a microreactor of our design (see S1 and S2) and its performance has been compared to that of the conventional batch methodology. Our findings prove that flow is more than capable of performing as well, and if not, even better than its batch counterpart, thus illustrating that continuous flow is an exciting new enabling technology that is necessary in furthering the evolution of modern day chemistry.

## Results and discussion

The full synthetic strategy of l-proline derivative **2** from fluorenone is described in the supporting information (SI). The final important step is the electrochemical oxidation of l-proline derivative **1**, which has had reaction conditions screened via two methods. Firstly, in batch, **1** was dissolved in a solution of NaOMe in MeOH and stirred in an undivided electrochemical cell. For reactions performed at − 30 °C, the cell was submerged in a bath of dry ice in acetonitrile. Secondly, in continuous flow, **1** was dissolved in MeOH only, unless that reaction was screened with the supporting electrolyte (NaOMe). The solution was put into a syringe and injected into the electrochemical microreactor by a syringe pump.

**Figure 4:**
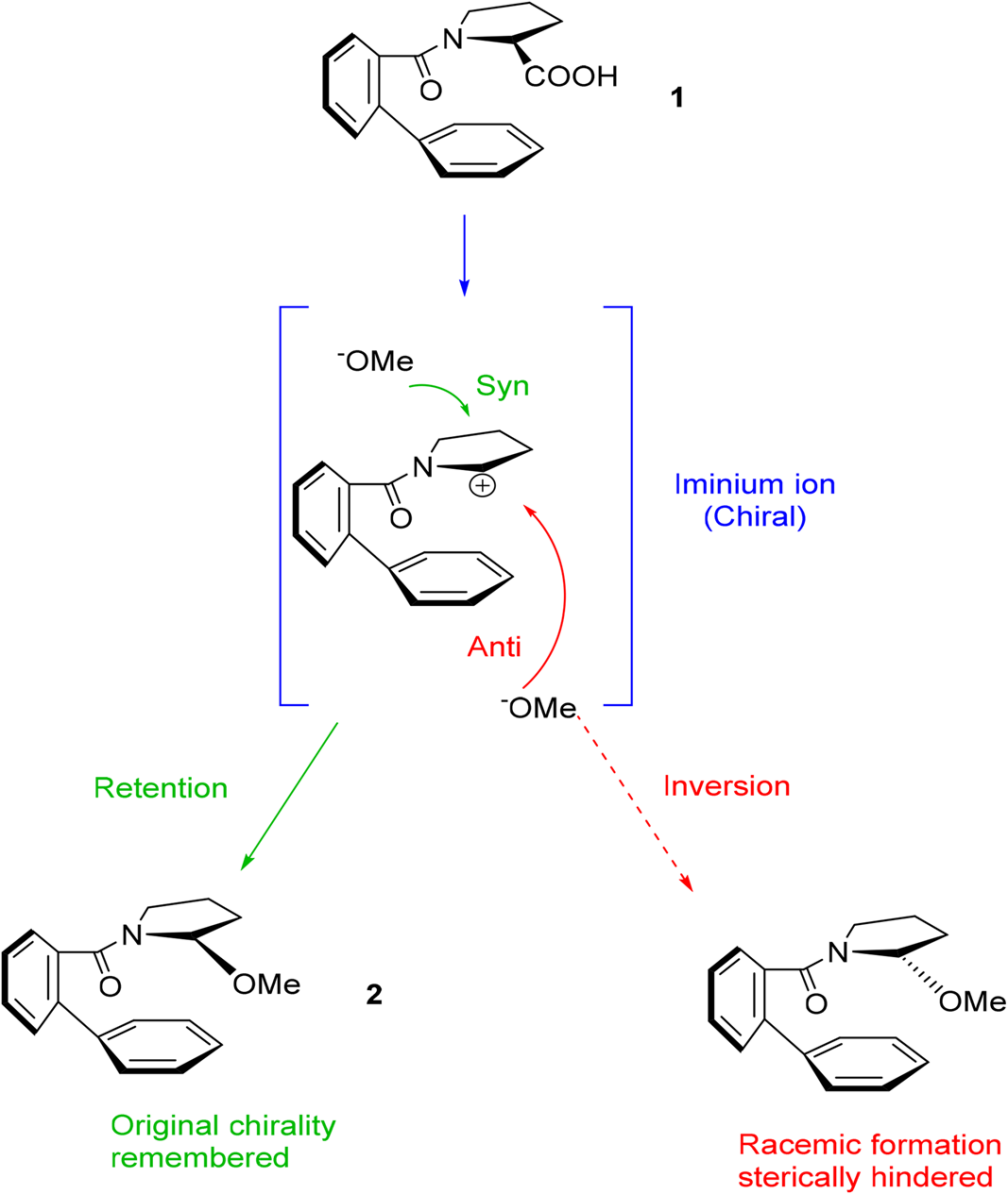
Mechanistic illustration of the MOC pathway, displaying two possible nucleophilic attack routes, for an *N*-benzoylated l-proline derivative undergoing an oxidation reaction.

Due to the close similarity between the compounds created by Matsumura et al.^36^ and ours, the reaction in Fig. 4 is applicable as a valid representation of our l-proline derivative^28^.

In order to increase the selectivity of the reaction so that the *R* isomer would be favoured, the requirements of MOC have been satisfied by attaching to the amino compound an *N*-protecting group with an additional phenyl group in the ortho position^35,37,38^. Its bulkiness is therefore, able to hinder nucleophilic attack from the *anti* side while restricting rotation so that the chiral iminium intermediate species is stable and in the same conformation as the initial material. The effect of this is evident from the batch electrolysis in which methoxide attacked from the more easily accessible *syn* face (under conditions of − 30 °C with platinum electrodes and methanol as the solvent).

**Figure 5:**
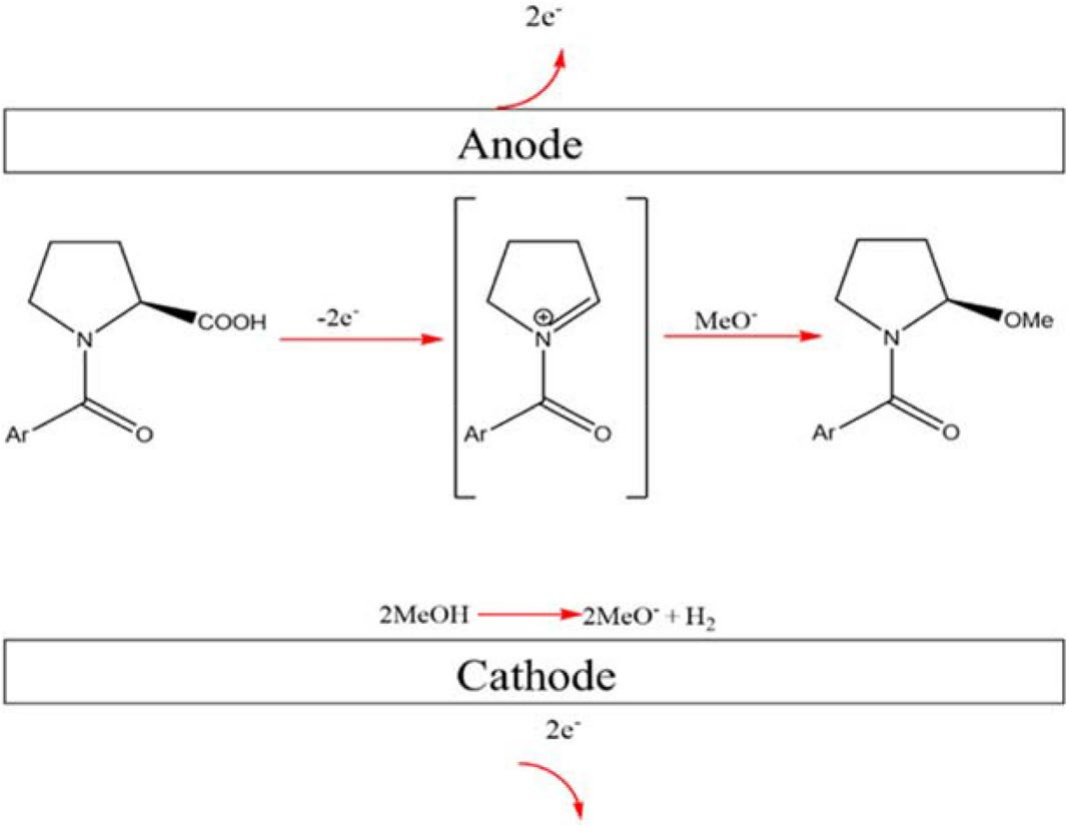
Schematic illustration of the Shono oxidation proceeding within the short interelectrode distance of the microreactor.

During electrolysis, the first step of the reaction facilitates a decarboxylation that results in the formation of a radical species. This step is followed by formation of an *N*-acyliminium ion, localised about the heteroatom which then allows subsequent nucleophilic attack to the carbon that underwent decarboxylation. Due to the assistance of this hetroatom (nitrogen), this whole scheme is classified as a non-Kolbe reaction. The final part of this reaction (nucleophilic attack to the *N*-acyliminium ions) is known as the Shono oxidation. Since the distance between the electrodes in the flow microreactor is very short (500 μm), this Shono oxidation may take place without the need of a supporting electrolyte and instead the solvent, methanol, acts as the charge carrier. The methoxy species becomes the nucleophile that will attack the *N*-acyliminium ion (Fig. 5).

### Batch electrolysis results

Preliminary screening of the reaction parameters were conducted in a batch electrochemical cell (results shown in Table S1)^39,40^. To begin with, the charge passing through the system was investigated. Using Pt/ C electrodes it can be seen that a low charge produces a small amount of product (entry 1). However, a larger of a charge of 2 F/mol resulted in the decomposition of the starting material (entry 2) because 50 mA (2 F/mol) was too much for electrodes of dimensions 1 cm × 1 cm to handle (note: entries **1** and **2** have the same charge but a different current as the time to reaction completion was different for each). Nevertheless, it seems that by fine tuning the charge, a larger *ee* was able to be produced at room temperature, as shown in entries **5**–**6**. Entries **3**–**4**, again, display screening of an increased charge with Pt/ Pt electrodes. Here, however, a significant change was not observed although it was found to be possible to obtain the same enantioselectivity in different conditions with a better yield (entry **3**). On that note, reactions performed with platinum as both electrodes showed a clear domination over the use of graphite as an anode (entry **2** and **4**). Variation in electrolyte species was also investigated and sodium methoxide (entry **1**) was found to be optimal in comparison to tetrabutylammonium tetrafluoroborate, ^n^Bu_4_NBF_4_, (entry **8**) and in the case of no electrolyte being used, since the space between the electrodes is so large (in conjunction to the short interelectrode gap in the microreactor), there is no current and therefore, no product formation (entry **9**). The fact that the reaction is still able to proceed in ^n^Bu_4_NBF_4_ implies that the base (NaOMe) is not the source of methoxylation and that probably the actual nucleophile is the solvent.

The idea of this work takes inspiration from the aforementioned works by Matsumura et al.^35,36^ however, their optimal conditions were dependant on a low temperature. A disadvantage to the microreactor used in this work is that there is no control over temperature, thus to compare our flow results to their optimised *ee* value of 46% would be unfair, therefore, we have also replicated their conditions used at − 30 °C (entry **4**) and at room temperature (entry **7**). By increasing the temperature to that of room temperature there is a significant loss of *ee* of 10%, which now begs the question of how much of a problem will this be in flow? Overall, Table S1 illustrates that the choice of electrode material is important as well as the effect of temperature. Form here, three benchmark *ee* values can be taken as target values for the flow reactions: 46% from the value reported by Matsumura et al.^35^ 41% from entry **3** as our optimised *ee* value and 30% from entry **7** which represents the fair comparison of reaction conditions optimised, but at room temperature.

### Flow electrolysis results

Table 1 shows the screening of flow reactions with the use of platinum as the cathode and graphite as the anode. By increasing the current it can be seen that enantiomeric excess generally increases (entries **1**–**4**). The same is true for the case where platinum is used as both electrodes (Table 2), but in this case, the different electrode material clearly dominates over the case of the graphite anode. Both of these findings are in agreement with that of the batch reactions. Increasing the concentration of the starting material 1 was found to slightly decrease the enantiomeric excess (Table 3), although not by a great amount. As mentioned in the introduction it is possible to perform these continuous flow reactions without a supporting electrolyte, due to electrogenerated ions being formed from the solvent within the short distance between electrodes. The solvent itself, methanol, is considered a good conducting solvent and in this situation, can also provide methoxide ions after reduction take place at the cathode that are used for further functionalization at target cationic species. However, one can be added if so desired, as in the case of entry **5** (Table 1). Here, a catalytic amount of NaOMe was added to the solution, which in turn afforded the greatest increase in *ee* of 14%. This positive effect of using a catalytic amount of NaOMe stems from it acting as both an electrolyte and as an additional source of methoxide ions to the mixture that are directly available for target compound functionalisation. Not only did the electrolyte increase the *ee* of the conditions that had the lowest value, it did so to such an extent that all three benchmark values were beaten, including the optimised value from the literature.

**Table 1:** Continuous flow elecrochemical screening of current/ charge results with platinum as the cathode and graphite as the anode.

Entry	Current (mA)	Charge (F/mol)	Electrolyte	Conversion (%)	Yield (%)	ee (%)
1	20	1.2	None	7	2.6	34
2	30	1.8	None	20	9.0	34
3	40	2.4	None	25	10.2	39
4	50	3.0	None	20	5.0	38
5	20	1.2	NaOMe	9	4.7	48

Constant parameters: electrodes (Pt cathode/C anode), Temperature (rt), flow rate (0.05 ml/min) and concentration (0.05 M). General procedure: a solution of 1-([1, 1′-biphenyl]-2-carbonyl) pyrrolidine-2-carboxylic acid **1** in MeOH was formed and was injected in the microreactor. The current and the flow rate were fixed. ee value were evaluated by chiral HPLC analysis. Isolated yield: purification by TLC preparative (silica gel, *n*-hexane/EtOAc 1:2).

**Table 2:** Continuous flow elecrochemical screening of current/ charge reults with platinum as both the anode and cathode.

Entry	Current (mA)	Charge (F/mol)	Conversion (%)	Yield (%)	ee (%)
1	20	1.2	22	8.5	35
2	30	1.8	24	10.6	35
3	40	2.4	25	10.8	43
4	50	3.0	20	10.0	42
5	100	6.0	15	7.0	45

Constant parameters: electrodes (Pt cathode/Pt anode), temperature (rt), flow rate (0.05 ml/min), concentration (0.05 M) and electrolyte (none). General procedure is followed as given above.

**Table 3:** Continuous flow elecrochemical screening of concentration reults with platinum as both the anode and cathode.

Entry	Concentration (M)	Conversion (%)	Yield (%)	ee (%)
1	0.05	22	8.5	35
2	0.1	20	8.3	34
3	0.2	23	8.7	31
4	0.3	19	8.3	30

Constant parameters: electrodes (Pt cathode/Pt anode), Temperature (rt), current (20 mA), charge (1.2 F/mol), flow rate (0.05 ml/min) and electrolyte (none). General procedure is followed as given above.

Finally, these flow conditions were optimised by using platinum electrodes (both as anode and cathode), a concentration of 0.05 M, a catalytic amount of NaOMe and a charge of 2.2 F/mol (Table 4). The flow rate of these reactions was reduced to allow maximum conversion of the starting material, resulting in significant increase in yields and *ee* values while avoiding overoxidation and the generation of unwanted side products at more optimised flow rates.

**Table 4:** Continuous flow elecrochemical screening of flow rate results with platinum as both the anode and cathode.

Entry	Concentration (M)	Charge (F/mol)	Flow rate (ml/min)	Conversion (%)	Yield (%)	ee (%)
1	0.05	2.2	0.04	69	45	48
2	0.05	2.2	0.03	83	54	55
3	0.05	2.2	0.025	94	65	61
4	0.05	2.2	0.01	98	71	64

Constant parameters: electrodes (Pt cathode/Pt anode), temperature (rt), current (2.2 F/mol), concentration (0.05 M) and electrolyte (NaOMe, catalytic amount). General procedure is followed as given above.

## Experimental

### Synthesis of (*R*)-[1,1′-biphenyl]-2-yl(2-methoxypyrrolidin-1-yl) methanone, 2, in batch

In a dry system, ([1,1′-biphenyl]-2-carbonyl)- l-proline (**1**, 74 mg/0.25 mmol) was added to a dispersed solution of MaOMe (135 mg/25 mmol) in methanol (5 ml) in a 10 ml undivided cell. The cloudy white solution was brought to – 30 °C with dry ice in acetonitrile. The current was fixed and stirring was activated. A yellow solution was formed and purification by TLC preparative (silica gel, *n*-hexane/EtOAc 1:2) produced a clear colourless solution. The MeOH was then evaporated under vacuum to give white crystals^37,39,40^.

### Synthesis of (*R*)-[1, 1′-biphenyl]-2-yl (2-methoxypyrrolidin-1-yl) methanone, 2, in flow

([1, 1′-biphenyl]-2-carbonyl)-l-proline (**1**, 44 mg/0.15 mmol) was dissolved in methanol (3 ml) and injected into the microreactor. Flow rate and current were set and reaction proceeded at room temperature. Product was collected as a colourless liquid of mixed rotamers^39^. ^1^H NMR (500 MHz, CDCl_3_): δ 7.46–7.25 (m, 9H), 5.38 (br s, 0.2H, minor), 4.23 (d, *J* = 4.2 Hz, 0.8H, major), 3.47–3.42 (m, 1.7H), 3.28–3.22 (m, 1H), 2.70 (s, 2.3H, major), 1.79–1.35 (m, 4H). ^13^C NMR (126 MHz, CDCl_3_): δ 171.5 (minor), 170.5 (major), 139.9, 139.8, 138.3, 136.6, 136.0, 129.7, 129.6, 129.3, 129.1, 128.7, 128.6, 128.5, 128.4, 127.8, 127.7, 127.6, 127.5, 126.9, 89.8 (major), 87.1 (minor), 56.5 (minor), 54.6 (major), 46.6 (minor), 44.4 (major), 31.2, 22.4, 20.9. IR (neat): ν 3059, 3026, 2976, 2883, 2829, 2362, 2343, 1735, 1625, 1587, 1560, 1450, 1145, 1074, 931, 904, 835, 763 cm^−1^. FTMS (NSI): Exact mass calc. for C_18_H_19_NO_2_ [M^+^ H]^+^ : 282.14, Found 282.15.*ee*: 64%, determined by HPLC analysis: Chiral 2D: OD-H 5 μm (250 × 4.6 mm), n-hexane/ isopropanol 90:10, 1.0 mL/min, 20 °C, 254 nm, retention time major (*R*) isomer = 6.413 min, retention time minor (*S*) isomer = 8.233 min. ${\left[\alpha \right]}_{D}^{20}$= − 0.96° cm^3^/g dm (c = 0.69, CHCl_3_).

## Conclusion

From production and small l-scale work performed in laboratories to scale-up to for industrial level applications, microreactors have evolved for the purposes of reaction optimisation, to obtaining kinetic and mechanistic information^4,41^, and have attracted enough attention to make this a hot topic that is hoped, at least for industrial purposes, to overtake the use of the batch methodology. Electrochemical oxidations have been performed in batch and flow, with and without the use of a supporting electrolyte. Dominant conditions were found when both electrodes used platinum and in general, continuous flow *ee* values increased with current and the addition of catalytic amount of an electrolyte. By comparing batch to flow, in every fair comparison, flow was able to outperform conventional batch methodology, and in some of the unfair comparisons, with flow being conducted with a temperature disadvantage, flow still showed a greater performance. In science we are constantly looking to advance ourselves and here we have illustrated the potential of continuous flow to be the next contender to revolutionise (organic) chemistry in a similar way that the invention of chromatography did over 60 years ago.

## Supplementary information

Supplementary Information.
