# Author Correction: Memory of chirality in a room temperature flow electrochemical reactor

**DOI:** 10.1038/s41598-022-15970-5

**Published:** 2022-07-11

**Authors:** Tomas Hardwick, Rossana Cicala, Thomas Wirth, Nisar Ahmed

**Affiliations:** 1 School of Chemistry, Cardiff University, Main Building, Park Place, Cardiff, CF10 3AT UK; 2 National Graphene Institute, University of Manchester, Oxford Road, Manchester, M13 9PL UK; 3 International Centre for Chemical and Biological Sciences, HEJ Research Institute of Chemistry, University of Karachi, Karachi, 75270 Pakistan

Correction to: *Scientific Reports* 10.1038/s41598-020-73957-6, published online 06 October 2020

An investigation by Cardiff University has concluded that Thomas Wirth was omitted from the author list in the original version of this Article. The author list now reads

“Tomas Hardwick, Rossana Cicala, Thomas Wirth & Nisar Ahmed”

The Author Contributions section now reads:

“T.H. and R.C. performed compound syntheses, batch and flow reactions, analysed results, wrote manuscript, T.W. designed and supervised the project, N.A. designed, wrote manuscript and supervised the project.”

The Competing Interests section is unchanged.

Thomas Wirth has agreed to this correction. Tomas Hardwick and Nisar Ahmed disagree with this correction. The Editors were not able to obtain the current contact details for Rossana Cicala.

The original Article and accompanying Supplementary Information file have been corrected.

