# Supplementary material for: Memory of chirality in a room temperature flow electrochemical reactor
Source: Sci Rep. 2020 Oct 6;10:16627. doi: 10.1038/s41598-020-73957-6 (PMC7539001; doi:10.1038/s41598-020-73957-6)
Supplement: Supplementary file 1 — Supplementary Information. [file 41598_2020_73957_MOESM1_ESM.docx]

**Supporting Information**

**Memory of Chirality in Room Temperature Flow Electrochemical Reactor**

Tomas Hardwick1,2, Rossana Cicala^1^, Thomas Wirth^1^ & Nisar Ahmed* 1,3

1School of Chemistry, Cardiff University, Main Building, Park Place, Cardiff, CF10 3AT, UK

2National Graphene Institute, University of Manchester, Oxford Road, Manchester, M13 9PL, UK

^3^International Centre for Chemical and Biological Sciences, HEJ Research Institute of Chemistry, University of Karachi, Karachi 75270, Pakistan

*E-mail: [nisarhej@gmail.com](mailto:nisarhej@gmail.com), AhmedN14@cardiff.ac.uk

**General** – The reactions were performed using standard laboratory equipment. All air sensitive reactions were carried out under a nitrogen atmosphere using oven dried glassware. All reactions were stirred using a stirrer plate and a magnetic stirrer bar and heating if necessary over a hotplate with a temperature probe control and adapted heating block. Lower temperatures were achieved using ice/water bath (0°C) and dry ice/acetonitrile bath (-30 °C). Buchi Rotavapors were used for solvent evaporations (reduced pressure up to 15 mbar) and a high vacuum apparatus was used to further dry the products. Dry THF, toluene and CH_3_CN were collected from a solvent purification system (SPS) from the company MBRAUN (MB SPS-800). Dry CH_2_Cl_2_ was distilled over calcium chloride. Other dry solvents as well as chemicals were purchased from Sigma Aldrich and were used without any further purification. Melting points were recorded on a Gallenkamp variable heater with samples in open capillary tubes.

**NMR Spectroscopy** - ^1^H NMR and ^13^C NMR spectra were recorded on a Bruker Fourier 300, a Bruker DPX 400, 500 and 600 apparatus and referenced to the residual proton solvent peak (^1^H: CDCl_3_, δ 7.26 ppm; CD_3_CN, δ 1.94 ppm; (CD_3_)_2_SO, δ 2.50 ppm; (CD_3_)_2_ CO), δ 2.05 ppm) and solvent ^13^C signal (CDCl_3_, δ 77.16 ppm; (CD_3_)_2_SO, δ 39.52 ppm; CD_3_)_2_ CO), δ = 29.84, 206.26 ppm). Chemical shifts, δ, were reported in ppm downfield of tetramethylsilane (δ = 0 ppm), multiplicity (s = singlet, d = doublet, t = triplet, q = quartet, quin = quintet, sex = sextet, hep = septet, m = multiplet, b = broad) and coupling constants (*J*) in Hertz.

**Infrared Spectroscopy**

IR spectra were recorded on a Shimadzu FTIR IR Affinity-1S apparatus with neat substance between NaCl disks. Wavenumbers are quoted in cm^–1^.

**Mass Spectrometry**

Mass spectrometric measurements were performed by the EPSRC Mass Spectrometry Facility in Swansea University on a Waters Xevo G2-S and on a Thermo Scientific LTQ Orbitrap XL machine for high resolution mass spectroscopy (HRMS) or by R. Jenkins/ R. Hick/ S. Waller at Cardiff University on a Water LCR Premier XE-tof. Ions were generated by the atmospheric pressure ionisation techniques (APCI), Electrospray (ES), Electron Ionisation (EI) or nanospry ionisation (NSI). The molecular ion peaks values quoted for either molecular ion (M^+^), molecular ion plus hydrogen (M^+^H^+^), molecular ion plus ammonium ion (M^+^NH_4_^+^) or molecular ion plus sodium (M^+^Na^+^).

**Optical Rotation**

Optical rotations were measured with a SCHMIDT and HAENSCH UniPol L polarimeter at 20 °C in cuvette of 500 mm length with a sodium light (589.30 nm). HPLC grade chloroform was used to prepare the solution and the concentration is indicated in the experimental section.

**Chromatography**

TLC by thin-layer chromatography (TLC) was performed on precoated aluminium sheets of Merck silica gel 60 F254 (0.20 m) and visualised by UV radiation (254 nm).

The HPLC measurements were carried out on a Shimadzu apparatus. The different modules were SIL-10ADVP (autoinjector), LC-10ATVP (liquid chromatograph), FCV-10ALVP (pump), DGU-14A (degasser), CTO-10ASVP (column oven), SCL-10AVP (system controller) and SPD M10A (diode array detector). The solvents used were n-hexane and 2-propanol and were bought from Fischer scientific as HPLC grade. The chiral column used for the separation of the enantiomers was Daicel Chiralcel OD-H (0.46 cm Ø x 25 cm).

**Synthesis of Reagents**

The full synthetic strategy of L-proline derivative **2** from fluorenone is described (ref in main text: 41,42). As a summation, a colourless mixture of KOH in xylene was initially prepared and brought to a temperature within the range of 170-180°C. Upon addition of yellow crystals of fluorenone, over a time period of 30 minutes, the colour of the solution changed from yellow to red/ orange after the first hour of stirring. A solvent system of xylene was able to yield the corresponding carboxylic acid in 76% yield, which is in contradiction to the use of toluene, in which no reaction took place at all. The reaction was also able to proceed at a reduced temperature range of 150-170°C, however, this occurred with a much lower yield of about 29%. It was also observed that a temperature below that of the above would not form the product. Under optimal conditions the reaction was able to afford the product as white crystals in 80% yield. The second step of the synthesis involved the chlorination of the carboxylic acid. SOCl_2_ and DMF were used to perform the well-known Vilsmeier-Haack reaction, generating the corresponding chlorinated organocatalyst as a reactive intermediate that would then be able to interact with the carboxylic acid moiety to afford the respective acid chloride as a yellow oil in 77% yield. This product is sensitive to moisture and therefore, the reaction was performed under inert, anhydrous conditions, with the addition of a nitrogen balloon at the top of the reflux apparatus. The third step of this synthesis was the addition of L-proline to the acid chloride. Since L-proline exists in its zwitterion form in solution, NaOH was used to increase the nucleophilicity of the nitrogen atom. From here, the lone pair was able to attack the carbonyl and replace the chloride. Finally, HCl was added to precipitate the L-proline derivative **1** as a white powder in 53% yield. It should be noted that for the first three steps no purification was performed and instead the crude product were used for the following step because it was found that too much of the product was lost in the column, i.e. the final step with purification yielded the product **1** in 15-17%. The final step is the electrochemical oxidation of L-proline derivative **1**, which has had reaction conditions screened via two methods. Firstly, in batch, **1** was dissolved in a solution of NaOMe in MeOH and stirred in an undivided electrochemical cell. For reactions performed at -30^o^C, the cell was submerged in a bath of dry ice in acetonitrile. Secondly, in continuous flow, **1** was dissolved in MeOH only, unless that reaction was screened with the supporting electrolyte (NaOMe). The solution was put into a syringe and injected into the electrochemical microreactor by a syringe pump.

**Stepwise synthesis is explained as follow:**

**Scheme S1**: Step one of synthetic strategy; fluorenone to [1,1’-biphenyl]-2-carboxylic acid.

**Synthesis of [1,1’-biphenyl]-2-carboxylic acid**: Potassium hydroxide (3.1g/ 0.056 mol) and xylene (30 ml) were added in a 100 ml two-necked flask and heated under reflux to 170-180 ^O^C, with the use of an oil bath to maintain constant temperature. During which time magnetic stirring was activated and the potassium hydroxide was dispersed. Over the next 30 min fluorenone (5g/ 0.028 mol) was added in four parts, after which the solution was stirred for 2h. After 1h a red/orange colour was observed. Water was added to stop the reaction and the product salt dissolved. The solution turned from a pinkish colour to yellow. Solution was allowed to stand for 30 min, separate and extracted with xylene in a separatory funnel. The aqueous layer was acidified to pH 1-2 with hydrochloric acid (2M). The crystals were filtered under gravity and dried in air. White crystals were obtained in 80% yield. ^1^H NMR (300MHz, (CD_3_)_2_ SO): δ (ppm) = 12.80 (s, 1*H*), 7.75 (dd, *J* = 7.6, 1.2 Hz, 1*H*), 7.57(dt, *J* = 7.5, 1.5 Hz, 1*H*), 7.40 – 7.32 (m, 7*H*); ^13^C (75 MHz, CDCl_3_): δ 173.78, 143.59, 141.29, 132.36, 131.48, 130.54, 129.65, 128.74 (2), 128.37 (2), 127.63, 127.48. m.p.: 110-114 °C. IR (neat): ν 3400, 3064, 2980, 1680, 1400, 1267, 1008, 918, 796, 635 cm^-1^. MS (EI): Exact mass calc. for C_13_H_10_O_2_ [M+ H]^+^ : 199.22, Found 199.07.

**Scheme S2**: Step two of synthetic strategy; [1,1’-biphenyl]-2-carboxylic acid to [1,1'-biphenyl]-2-carbonyl chloride.

**Synthesis of [1,1'-biphenyl]-2-carbonyl chloride**: Carboxylic acid (2.8g/ 0.014 mol) was dissolved in toluene (40 ml) in a 100 ml two-necked flask, and then thionyl chloride (6.1 ml/ 0.084 mol) and 2-3 drops of DMF were added. The solution was heated to 80 ^O^C and stirred under reflux for 3h. A balloon filled with nitrogen was placed at the top of the condenser. The pale yellow solution was evaporated under vacuum to afford a yellow oil product of 77% yield. ^1^H NMR (300MHz, (CD_3_)_2_ SO): δ (ppm) = 7.72 (dd, J= 7.6, 1.4 Hz, 1H) 7.54 (dt, J = 7.5,1.5 Hz, 1H), 7.35 - 7.27 (m, 7H); ^13^C NMR (75 MHz, (CD_3_)_2_ SO): δ 169.80, 141.30, 141.17, 132.52, 131.15, 130.77, 129.39, 128.60 (2), 128.39 (2), 127.54, 127.44. IR (neat): v 3061, 3028, 1778, 1471, 1188, 856, 769, 698 cm^-1^. MS (TOF): Exact mass calc. for C_13_H_9_ClO [M+ H]^+^ : 217.0344, Found 217.0371.

**Scheme S3**: Step three of synthetic strategy; compound [1,1'-biphenyl]-2-carbonyl chloride to ([1, 1’-biphenyl]-2-carbonyl)-L-proline**, 1**.

**Synthesis of ([1, 1’-biphenyl]-2-carbonyl)-L-proline**: to an ice-cooled solution of sodium hydroxide (0.4g/ 0.0098 mol) in water (8 ml), L-proline was added (0.56g/ 0.0049 mol). The solution was stirred for 2h at 0 ^O^C in an ice bath. THF (8 ml) was added, followed by acyl chloride (1.06g/ 0.0049 mol) and stirred overnight at r.t. The pale yellow solution was washed with diethyl ether, and the aqueous layer was removed in a separatory funnel. This aqueous layer was acidified to pH 1-2 with 10% HCl and the solution turned a milky-white colour with beige sticky mounds at the bottom. This was extracted with EtOAc (3 x 20 ml). The colourless aqueous layer was removed and the orange organic layer was washed three times with distilled water. This colourless aqueous layer was removed to leave the slightly white and cloudy organic layer, which was dried over MgSO_4_, filtered under gravity and evaporated under vacuum to afford the product as a white powder with 53% yield. ^1^H NMR (300 MHz, CDCl_3_): δ 7.50 – 7.24 (m, 9H), 4.24 (s, 1H), 3.51 – 3.36 (m, 1H), 3.31 – 3.20 (m, 1H), 1.89 – 1.58 (m, 1H), 1.40 (m, 1H).^13^C NMR (101 MHz, CDCl_3_): δ 170.75, 140.04, 136.21, 129.77, 129.49, 128.87, 128.76 (2), 128.72 (2), 128.62, 127.86, 127.72, 89.99,54.77, 44.58, 31.37, 21.12. m.p.:153-160. IR (neat): ν 3061, 3026, 2976, 2954, 2941, 2883, 2362, 1734, 1697, 1625, 1585, 1560, 1411, 1143, 1195, 912, 835, 777, 719 cm^-1^. MS (TOF): Exact mass calc. for C_18_H_18_NO_3_ [M+ H]^+^ : 294.12, Found 296.12. $\left[ \alpha\right]_{D}^{20}:$ - 1.13° cm^3^/ g dm (c = 0.67, CHCl_3_). R_f_: = 0.5 (silica gel, n-hexane/ EtOAc 1:2).

**Scheme S4**: Step three of synthetic strategy; compound **1** to **2**.

**Synthesis of (*R*)-[1,1'-biphenyl]-2-yl(2-methoxypyrrolidin-1-yl) methanone, 2, in batch**: In a dry system, ([1,1'-biphenyl]-2-carbonyl)-L-proline (**1**, 74 mg/ 0.25 mmol) was added to a dispersed solution of MaOMe (135 mg/ 25 mmol) in methanol (5 ml) in a 10 ml undivided cell. The cloudy white solution was brought to -30 ^O^C with dry ice in acetonitrile. The current was fixed and stirring was activated. A yellow solution was formed and purification by TLC preparative (silica gel, n-hexane/ EtOAc 1:2) produced a clear colourless solution. The MeOH was then evaporated under vacuum to give white crystals (ref in main text: 41,42)

**Batch Electrolysis Results:**

| Entry | Temperature  (^o^C) | Charge  (F/ mol) | Current  (mA) | Electrodes | Electrolyte | Yield  (%) | ee  (%) |
| --- | --- | --- | --- | --- | --- | --- | --- |
| 1 | -30 | 2 | 25 | Pt cathode  C anode | NaOMe | 1.3 | 18 |
| 2 | -30 | 2 | 50 | Pt cathode  C anode | NaOMe | Dec | No product |
| 3 | -30 | 1.2 | 10 | Pt cathode  Pt anode | NaOMe | 44 | 41 |
| 4 | -30 | 2 | 25 | Pt cathode  Pt anode | NaOMe | 15 | 40 |
| 5 | rt | 1.6 | 20 | Pt cathode  C anode | NaOMe | 4.7 | 20 |
| 6 | rt | 2.4 | 30 | Pt cathode  C anode | NaOMe | 23.7 | 28 |
| 7 | rt | 2 | 25 | Pt cathode  Pt anode | NaOMe | 47 | 30 |
| 8 | -30 | 2 | 25 | Pt cathode  Pt anode | ^n^Bu_4_NBF_4_ | 10 | 21 |
| 9 | -30 | 2 | 25 | Pt cathode  Pt anode | None | Dec. | - |

**Table S1**: *Batch elecrochemical screening reults. Constant parameter: concentration* *(0.05M).* *General procedure: 1-([1,1’-biphenyl] -2-carbonyl) pyrrolidine-2-carboxylic acid* ***1*** *was added in the batch reactor and the temperature was brought to – 30°C using* acetonitrile*/dry ice. A solution of NaOMe in MeOH was formed and added into the flask. The current was fixed and the reaction mixture was monitored by using TLC plate (silica gel, n-hexane/EtOAc 20%). ee value were evaluated by HPLC analysis. Isolated yield: purification by TLC preparative (silica gel, n-hexane/ EtOAc 1:2).*

**Synthesis of (*R*)-[1, 1’-biphenyl]-2-yl (2-methoxypyrrolidin-1-yl) methanone, 2, in flow**: ([1, 1’-biphenyl]-2-carbonyl)-L-proline (**1**, 44 mg/ 0.15 mmol) was dissolved in methanol (3ml) and injected into the microreactor. Flow rate and current were set and reaction proceeded at room temperature. Product was collected as a colourless liquid of mixed rotamers. ^1^H NMR (500 MHz, CDCl_3_): δ 7.46 – 7.25 (m, 9H), 5.38 (br s, 0.2H, minor), 4.23 (d, *J* = 4.2 Hz, 0.8H, major), 3.47 – 3.42 (m, 1.7H), 3.28 – 3.22 (m, 1H), 2.70 (s, 2.3H, major), 1.79 – 1.35 (m, 4H).^13^C NMR (126 MHz, CDCl_3_): δ 171.5 (minor), 170.5 (major), 139.9, 139.8, 138.3, 136.6, 136.0, 129.7, 129.6, 129.3 , 129.1, 128.7, 128.6, 128.5, 128.4, 127.8, 127.7, 127.6, 127.5, 126.9, 89.8 (major), 87.1 (minor), 56.5 (minor), 54.6 (major), 46.6 (minor), 44.4 (major), 31.2, 22.4, 20.9. IR (neat): ν 3059, 3026, 2976, 2883, 2829, 2362, 2343, 1735, 1625, 1587, 1560, 1450, 1145, 1074, 931, 904, 835, 763 cm^-1^. FTMS (NSI): Exact mass calc. for C_18_H_19_NO_2_ [M^+^ H]^+^ : 282.14, Found 282.15.*ee*: 64%, determined by HPLC analysis: Chiral 2D: OD-H 5 μm (250 x 4.6 mm), n-hexane/ isopropanol 90:10, 1.0 mL/ min, 20 °C, 254 nm, retention time major (*R*) isomer = 6.413 min, retention time minor (*S*) isomer = 8.233 min.

$\left[ \alpha\right]_{D}^{20}$ = - 0.96° cm^3^/ g dm (c = 0.69, CHCl_3_). (ref in main text: 42)

**Microreactor setup**

In this work we have constructed what is classified as a parallel plate-to-plate chip type electrochemical microreactor (**Figure S1**). It consists of two aluminium bodies (75 x 75 x 25 mm), both with eight holes around the perimeter for screws to be fitted as well as a central hole in the centre to connect a copper wire to the electrode material within. One of these bodies has two extra holes cut into it that act as the outlet and inlet ports with HPLC fittings for secure flow of the reaction. On the opposite side there is a square compartment (50 x 50 mm) in which the electrode material can be placed onto a rigid copper or Teflon plate and connected to the copper wire and hence the power supply. However, for thicker electrode materials, such as graphite (2.5 mm thick), there is no need for a rigid plate, so an inexpensive stainless steel thin sheet was welded to a copper wire (Figure 1G, H). An FEP (fluorinated ethylene propylene) film spacer separates the anode and cathode, which has a snaking flow channel cut into it. The interelectode distance therefore depends on the thickness of the spacer (500 µm used in this work).


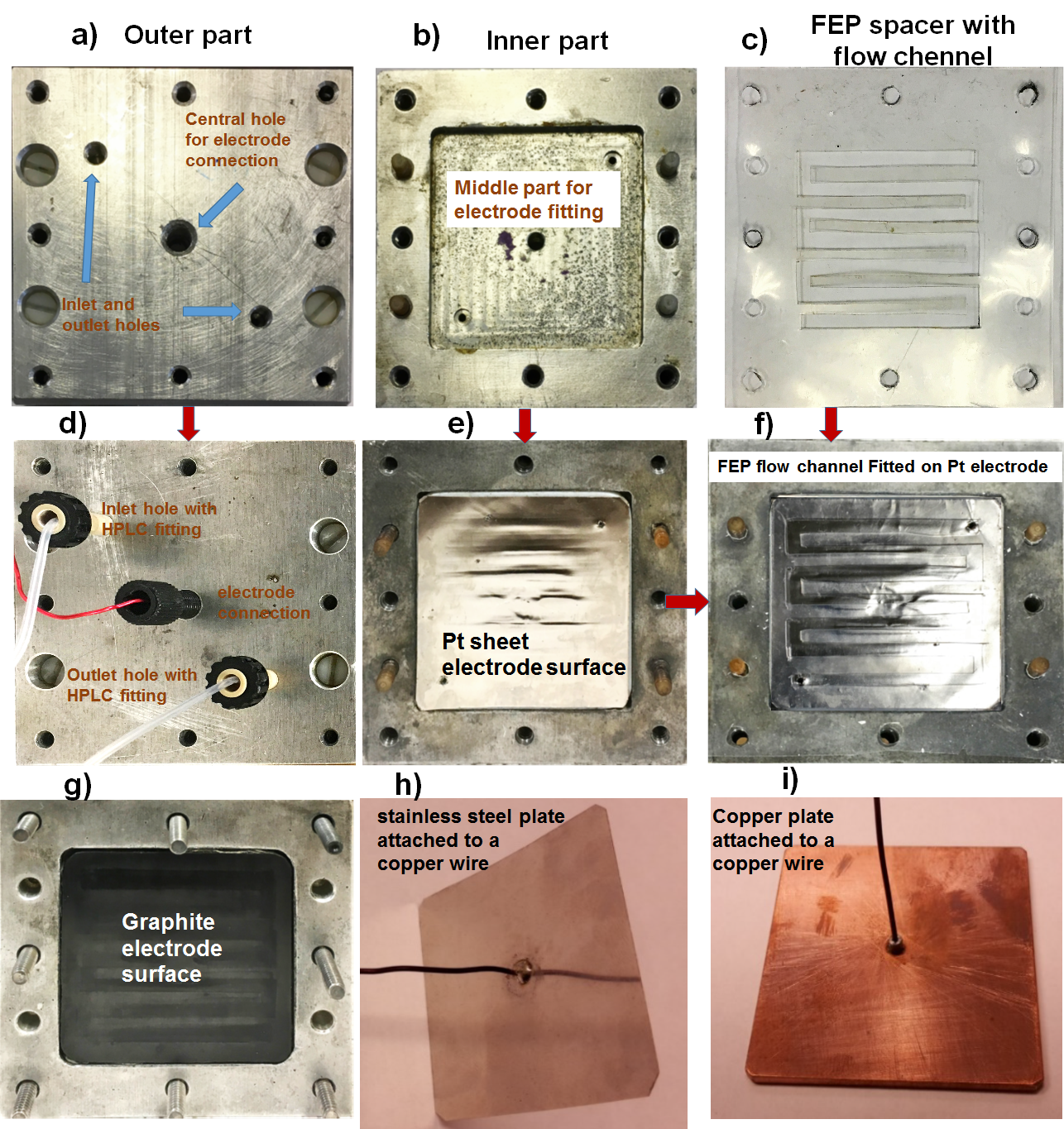


**Figure S1**. Electrochemical flow reactor. a) outer part of aluminium body; b) inner part of aluminium body; c) FEP spacer with flow channel d) outer part with HPLC fittings; e) Pt electrode fitted in middle part of aluminium body on teflon plate (other choice is copper plate); f) FEP flow channel fitted on Pt electrode; g) graphite electrode fitted in middle part of aluminium body on stainless steel plate; h) stainless steel plate welded to a copper wire; i) Copper plate welded to a copper wire.

**
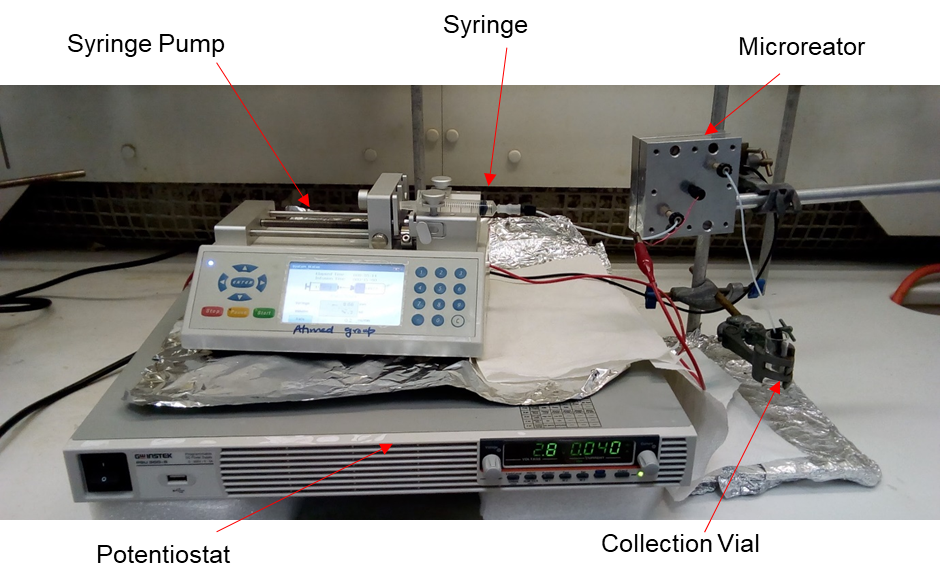
**

**Figure S2**: Photograph of flow setup.

**Spectra
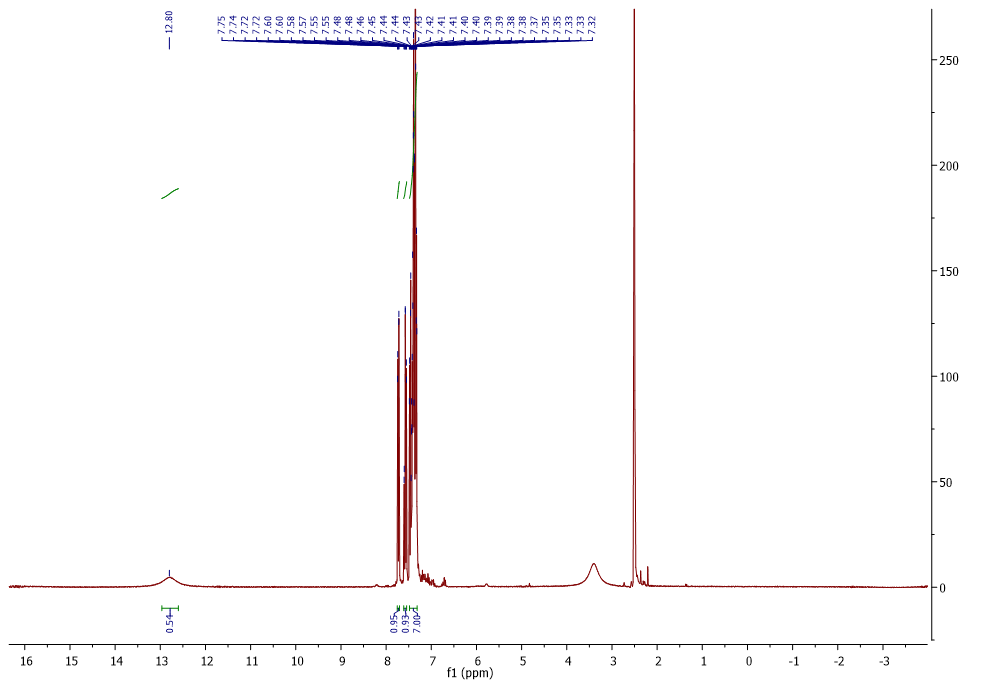

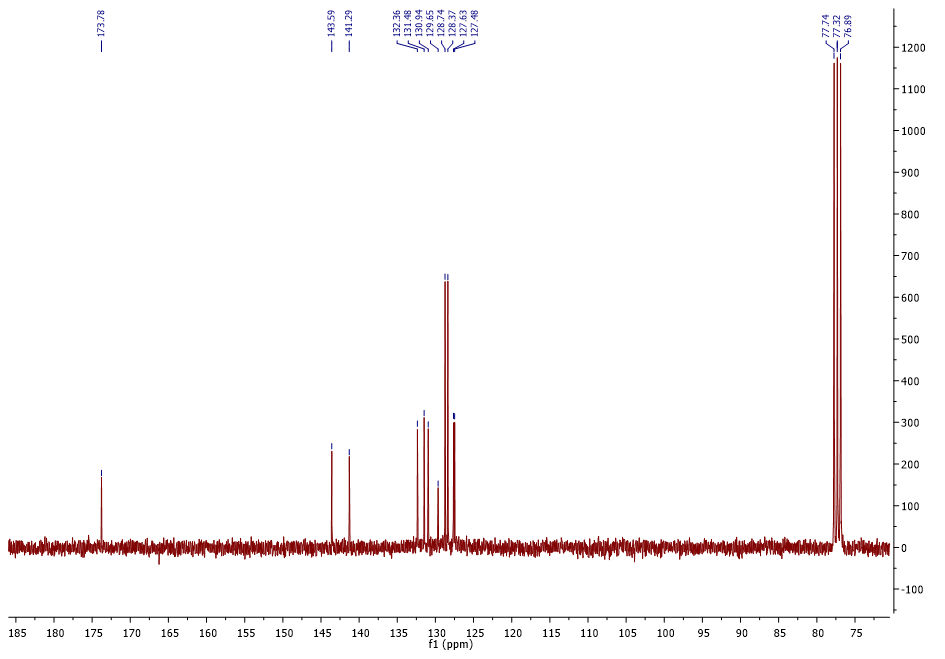
**

**Figure S3**: ^1^H NMR and ^13^C NMR spectra of [1, 1’-biphenyl]-2-carboxylic acid.


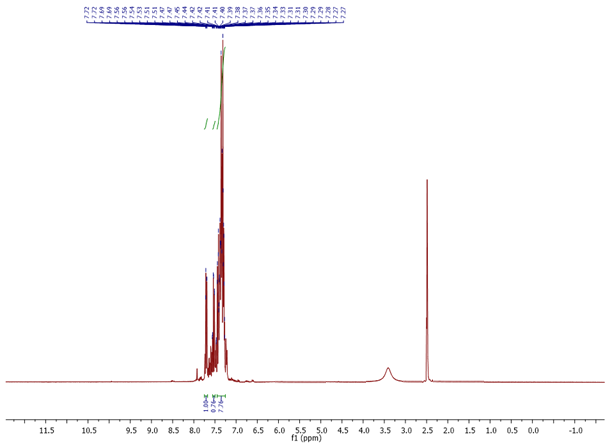


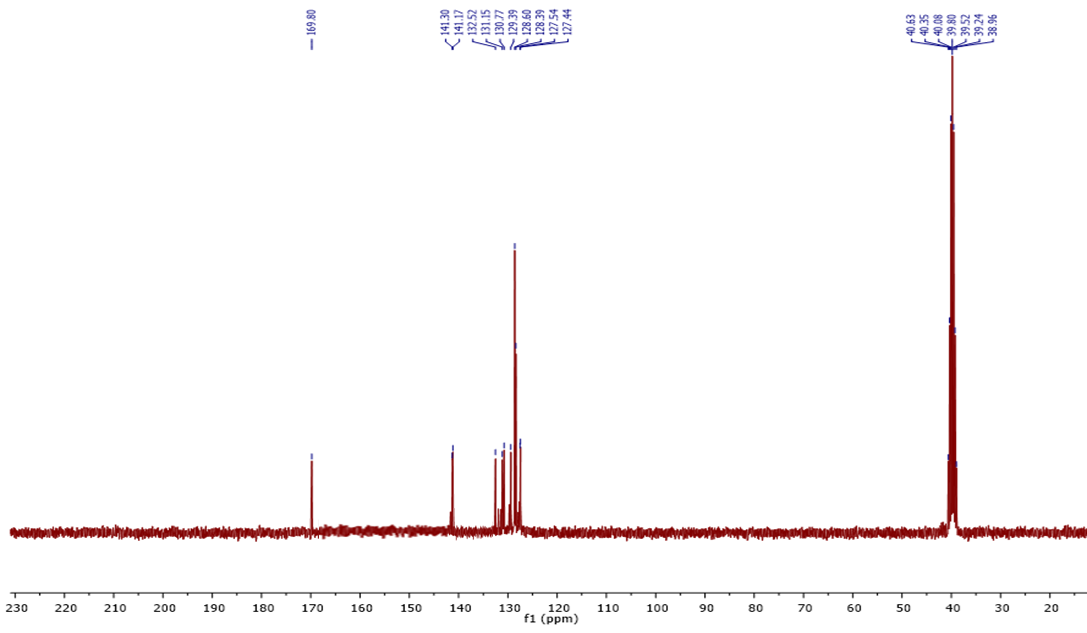


**Figure S4**: ^1^H NMR and ^13^C NMR spectra of [1, 1'-biphenyl]-2-carbonyl chloride.


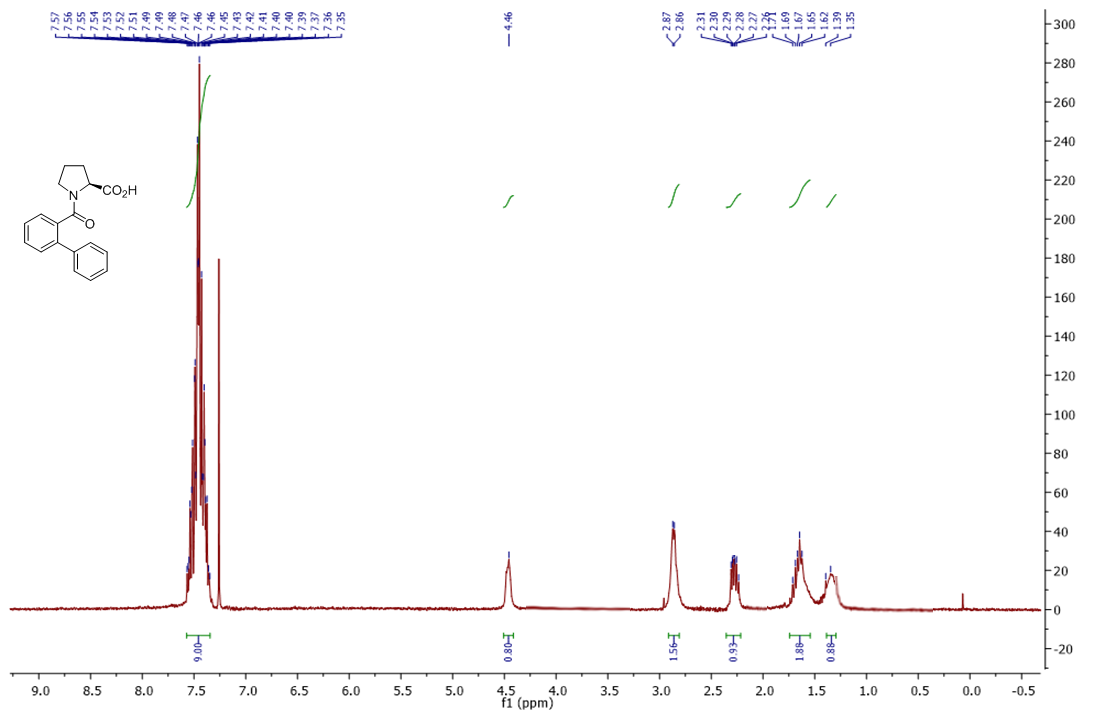


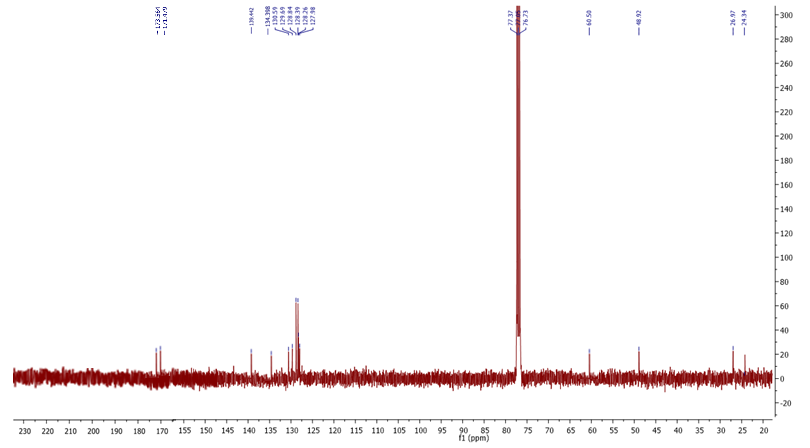


**Figure S5**: ^1^H NMR and ^13^C NMR spectra of ([1, 1'-biphenyl]-2-carbonyl)-L-proline, **1**.

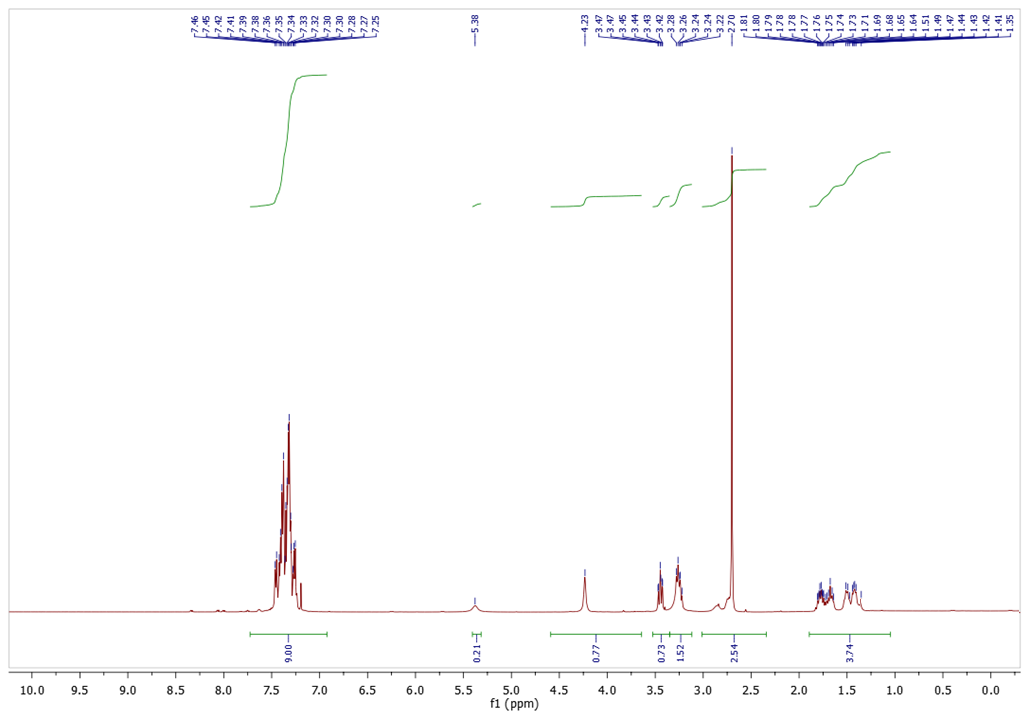


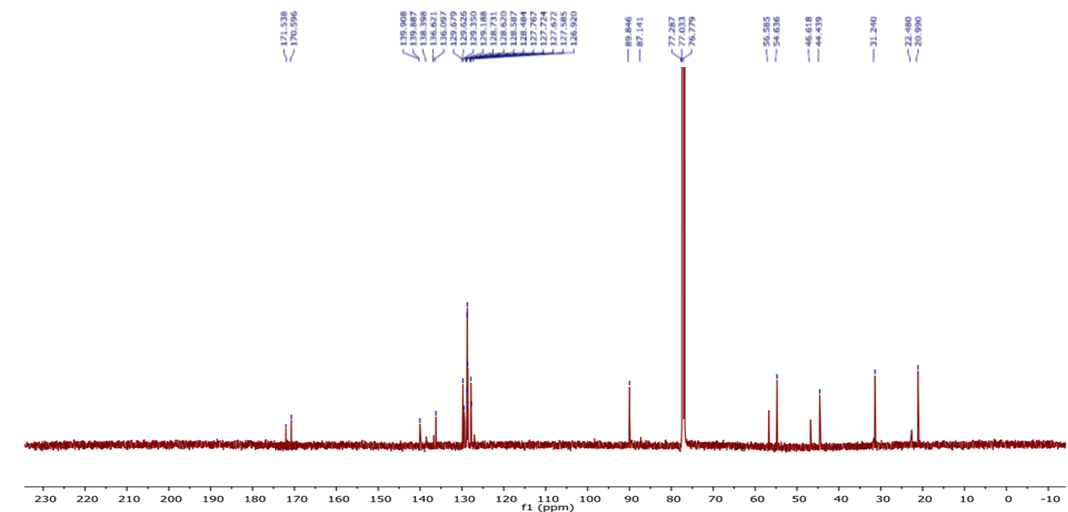


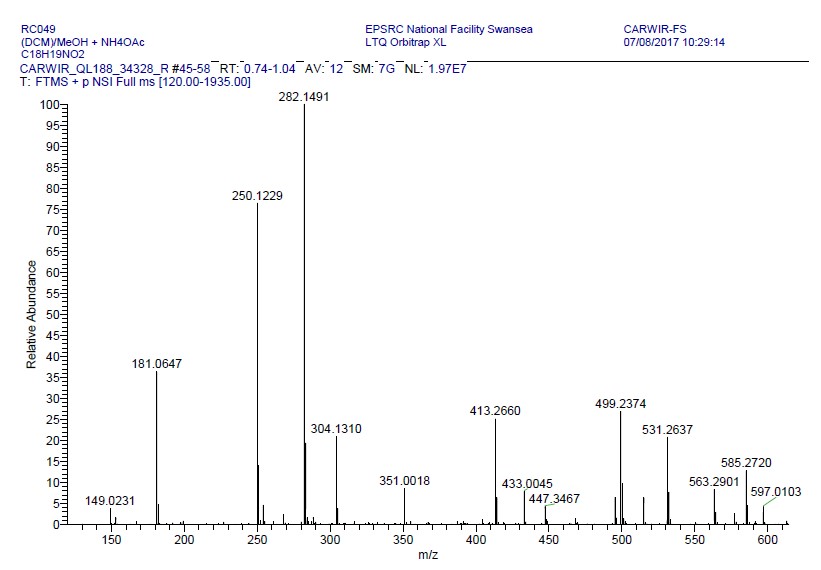


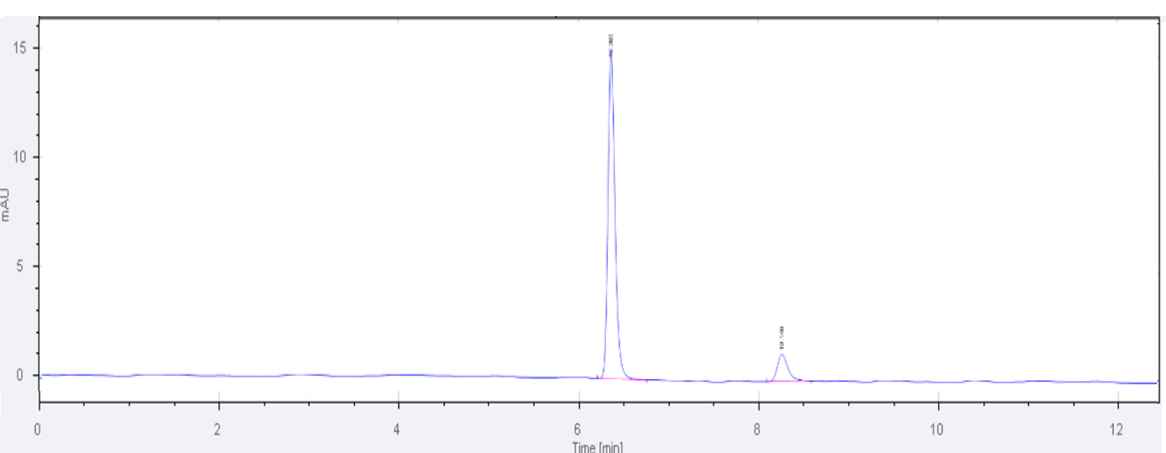


| **Peak** | **Retention time (min)** | **Area (%)**  Sample b |
| --- | --- | --- |
| 1 | 6.413 | 81.99% |
| 2 | 8.287 | 18.01% |
| Total |  | 100% |

*Compound* ***2****: ee* = 64%

**Figure S6**: ^1^H NMR, ^13^C NMR, MS and HPLC spectra of (*R*)-[1, 1’-biphenyl]-2-yl (2-methoxypyrrolidin-1-yl) methanone, **2**.
